# Recalibrating populism measurement tools: Methodological inconsistencies and challenges to our understanding of the relationship between the supply- and demand-side of populism

**DOI:** 10.3389/fsoc.2022.970043

**Published:** 2022-10-14

**Authors:** José Javier Olivas Osuna, José Rama

**Affiliations:** ^1^Department of Political Science and Administration, National Distance Education University (UNED), Madrid, Spain; ^2^LSE IDEAS, The London School of Economics and Political Science, London, United Kingdom; ^3^Department of Political Science and International Relations at Universidad Autónoma de Madrid, Madrid, Spain

**Keywords:** populism, methodology, political sociology, populist attitudes, political parties, supply-side populism, demand-side populism, populist discourses

## Abstract

The analysis of the congruence between the demand- and supply-side of populism is key to understand the relationship between citizens and populist parties, and to what extent this is mainly a “pull” or “push” phenomenon. Although the study of populism has experienced an unprecedented growth across social sciences during the last decade, research directly addressing this connection remains scarce. Moreover, most existing tools used to measure populism have not been created paying much consideration to their compatibility with those applied in the other side of this demand-supply divide. This article critically revisits the influential Comparative Study of Electoral Systems (CSES) Module 5 dataset to illustrate shortcomings regarding current efforts to measure the demand- and supply-sides of populism. We show that according to CSES data the, often presumed, correspondence between “populist” attitudes and likelihood of voting for “populist parties” is only partial and country specific. But more importantly, we identify three main potential sources of such mismatch linked to instrumental issues: (i) problems with the choice, design and operationalization of attitudinal survey items; (ii) problems in the assessment of parties' populism; and (iii) instrument biases that make them more effective with some varieties of populism than with others. These methodological limitations are hindering our ability to settle longstanding theoretical debates concerning the correspondence between the demand- and supply-side, the relative centrality of attributes, and varieties of populism. Therefore, we invite scholars working in this field to update existing measurement tools, or develop new ones, considering the multidimensionality of this latent construct, the diversity of movements, and the need to apply consistent criteria and operationalization techniques when assessing degrees of populism in citizens and parties.

## Introduction

Populism is widely considered as one of the major challenges for liberal democracies (Kriesi et al., 2008; Müller, 2016; Caamaño and Bértoa, 2020; Juon and Bochsler, 2020) and an area of research that has grown rapidly within the fields of sociology and political science in the last decade.^1^ A variety of conceptualization and ontological approaches compete to capture this complex phenomenon (Berlin, 1968; Gidron and Bonikowski, 2013; Olivas Osuna, 2021).

According to these different interpretations of populism, several research strategies have been developed to assess and classify political parties and leaders according to their level of populism (e.g., Rooduijn and Pauwels, 2011; Polk et al., 2017; Bernhard and Kriesi, 2019). This is what, following a microeconomics analogy, is usually known as the “supply-side” in the study of populism. Similarly, another strand in the literature has focused on designing items and scales to assess its “demand-side”, i.e., populist attitudes and beliefs manifested or felt among citizens or voters (e.g., Hawkins et al., 2012; Akkerman et al., 2014; Schulz et al., 2018).^2^

Presumably,supply- and demand-sides should be connected in this “populist marketplace” (Neuner and Wratil, 2022). For instance, if we assume that left-wing voters are more prone to support political parties displaying a left-wing ideology and proposing left-leaning policies, it seems logical to expect voters with a populist understanding of political dynamics to tend to endorse parties that uphold populist discourses and ideas. Likewise, it is reasonable to assume that the parties which are considered populist target individuals who share populist worldviews and attitudes, and tailor their messages accordingly.

While several authors show that attitudes and beliefs commonly associated to populism are strong among prospective voters of populist parties (Akkerman et al., 2017; Van Hauwaert and van Kessel, 2018, p. 72; Marcos-Marne, 2021; Mazzoleni and Ivaldi, 2022), other recent analyses find that this relationship only holds true in some countries (Jungkunz et al., 2021), reveal limitations to the explanatory power of some populist attitudes on vote choice (Neuner and Wratil, 2022), a significant impact of positive and negative partisanships on populist voters' behavior (Koch et al., 2021) and even that some populist parties attract people with elitist attitudes (Akkerman et al., 2014). Moreover, recent studies prove the variety of psychosocial traits displayed by individuals who support populist parties, in terms of ideological orientation (Vasilopoulos and Jost, 2020), attitudes toward outgroups (Pellegrini, 2022) and personality (Fatke, 2019) among other. Thus, “Do *populist voters* support *populist parties*?” remains one of the most important research questions in this field of study.

However, to settle this debate it is necessary to previously tackle some methodological questions such as: Are we consistently measuring populist attitudes? Are we classifying parties and leaders as populist or non-populist in a valid and reliable way? Are we applying equivalent criteria in both sides of the phenomenon? In sum, do we have the appropriate instruments to measure demand- and supply-sides of populism in a congruent fashion? Extant methodological articles in this incipient area of research have largely focused on assessing and comparing the validity of demand-side measurement tools (Castanho Silva et al., 2020; Van Hauwaert et al., 2020; Wuttke et al., 2020). This paper confirms some of the concerns raised by these studies, but additionally reveal other issues related to the assessment of the supply-side, the geographic context, and varieties of populism that invite to a more profound rethinking of what it is being currently done in terms of populism measurements.

This article explores to what extent the potential lack of congruence between the supply- and demand-side of populism can be attributed to the tools used to measure it. Firstly, we provide an overview of the most prominent approaches and instruments in the literature and their contribution to better understand the demand and supply aspects of this phenomenon.

Secondly, as a preliminary step to assess the congruence between the techniques used to measure both sides of populism, we test in seventeen countries—eleven European and six non-European—whether the probability of supporting a populist party is directly correlated with populist attitudes. We draw from the Comparative Study of Electoral Systems (CSES) Module 5 (Hobolt et al., 2016) dataset that measures, via large-n survey analysis, three dimensions of populist attitudes—i.e., attitudes toward elites, majority rule and democracy, and out-groups,—and the degree of populism in political parties based on country expert surveys. Using a variety of analytical approaches—such as, country by country logistic and linear regressions, average marginal effects, and country fixed effects estimations—we show that, while there is statistically significant congruence between the supply- and demand-side of populism in countries such as Austria, Germany, Norway, and Italy, the correspondence is only partial in France and Lithuania and null in other cases, such as Brazil, Korea, and Greece.

Finally, and more important, based on a second round of statistical analysis we demonstrate that the observed mismatches in the populist marketplace can be, to some extent, explained by methodological issues regarding the selection of definitional dimensions, design of attitudinal items, and criteria in the assessment of populist parties. We discuss these findings against the backdrop of recent contributions to the literature on populism, that have also identified some theoretical and methodological shortcomings. The limitations revealed suggest the need to revisit and recalibrate the CSES Module 5 and other commonly used populism measurement tools. To establish whether the success of populism is rooted on citizens' attitudes, or if populist discursive frames activate certain reactions in voters, it is important to first ensure that we use coherent indicators to measure the demand- and supply-sides of populism, and test also alternative approaches, grounded on slightly different theoretical and methodological standpoints.

## Measuring populism

Populism cannot be consistently identified with a specific socio-economic group, type of policies or political ideology (Müller, 2016, p. 11–19). The large and increasing set of movements termed as populist, each of them with different characteristics (Mudde, 2004, p. 548–551), has contributed to the problem of conceptual stretching. To the extent that Rodrik (2018, p.12) defines populism as “a loose label that encompasses a diverse set of movements”. In this context of conceptual indeterminacy, it is not surprising that many authors (e.g., Rodrik, 2018; Hopkin and Blyth, 2019; Norris and Inglehart, 2019) have undertaken ambitious empirical studies about the emergence and success of populist parties without putting excessive emphasis on engaging in the debates about the conceptualization and assessment of populism (Olivas Osuna, 2021, p. 831). In the following two sub-sections we explore the instruments currently used to measure populist attitudes and the criteria followed to classify parties as “populist” and “non-populist”.

### Measuring voters' populist attitudes

Populism is a multifaceted phenomena and has been studied from different ontological standpoints. It is sometimes construed as an ideology (Mudde, 2004; Stanley, 2008); political strategy (Weyland, 2001; Barr, 2018); discursive logic of articulation (Laclau, 2005; Aslanidis, 2016), and performative style (Moffitt, 2016; Ostiguy et al., 2021). Despite the alleged conceptual indeterminacy and the differences in the interpretations of the *genus* of populism, most attempts to measure the demand-side of populism adopt an ideational approach and consider it as a “thin-centered” ideology that characterizes politics as a Manichean struggle between the will of the homogenous people and the corrupt elite (Mudde, 2004, p. 543; Hawkins and Kaltwasser, 2017, p. 3). Most of the proponents of this ideational approach, acknowledge that populism is a multidimensional phenomenon and that populist attitudes lie at the intersection of several of such dimensions (Castanho Silva et al., 2018; Hameleers and de Vreese, 2020; Wuttke et al., 2020).

One of the most influential, and widely used, instruments to measure populist attitudes is the scale designed by Akkerman et al. (2014), which is built upon the work of Hawkins et al. (2012) and captures with eight items, three broad dimensions of populism: (i) the notion of popular sovereignty, (ii) anti-elitism, and (iii) a Manichean worldview. This scale initially designed and tested empirically in the Netherlands has been later applied in different case studies (Spruyt et al., 2016; Meléndez and Rovira Kaltwasser, 2019; Zanotti and Rama, 2021), and cross-country surveys (Van Hauwaert and van Kessel, 2018). Following this line of research, Castanho Silva et al. (2020) propose a scale which expands the number of items but keeps the focus on the same three core dimensions and Van Hauwaert et al. (2020) suggest a refinement of the scale of Akkerman et al. (2014) by identifying the “best-three” performing items.

There are other relevant and slightly different approaches to the measurement of the demand-side of populism. For instance, Elchardus and Spruyt (2016) use a four-item scale that tries to capture different aspects of people centrism and anti-elitism, paying special attention to the sense of disconnect with experts and politicians. Oliver and Rahn (2016) propose a scale that focuses on anti-elitism, mistrust of experts and national affiliation. Schulz et al. (2018) use fifteen-item instrument to identify anti-elitism, popular sovereignty, and understanding of the people as being homogenous and virtuous. Finally, Hobolt et al. (2016) instrument to measure populism within the CSES Module 5 (further analyzed in the following sections) focuses on attitudes toward political elites, out-groups, representative democracy and majority rule.

### Measuring populism in political parties

Despite some divergences in terms of specific survey items, wording of questions, and dimensions included, overall, the abovementioned scales of populist attitudes follow largely similar methodological and conceptual approaches (Castanho Silva et al., 2020; Van Hauwaert et al., 2020; Wuttke et al., 2020). However, there seem to be more diversity in terms of methodologies, as well as specific attributes and dimensions taken into consideration, in the instruments used to assess the supply-side of populism. On the one hand, some authors base their measurements on the analysis of textual material. For instance, Hawkins (2009), in his analysis of speeches from Latin American political leaders, introduces the *holistic grading* technique that requests coders, familiarized with the definition of populism, to interpret texts, and assign a single mark in a three-point scale 0 (“non-populist or pluralist”), 1 (“mixed”), or 2 (“populist”) (Hawkins, 2009, p. 1050). Among several other empirical works, this approach has inspired the Global Populism Database which covers 215 leaders in 66 countries (Hawkins et al., 2019).

Bernhard and Kriesi (2019, p. 1196) measure the degree of populism by analysing press releases issued by political parties in parliamentary elections in eleven European countries. They also use a classical content analysis approach but analyse three ideational subdimensions: people centrism, anti-elitism, and popular sovereignty. On the other hand, Pauwels (2011) adopts a computerized quantitative text analysis drawing on a dictionary-based approach. He analyses internally and externally oriented party literature by identifying words associated to populism—e.g., “the people”, “elite”, “establishment” and “corruption”—, and to other categories, such as conservative values, environmental issues, immigration, liberalism, progressive issues, nationalism, and law and order (Pauwels, 2011, p. 104–105). Rooduijn and Pauwels (2011) launched a similar computer-based study on party manifestos in four countries, and Di Cocco and Monechi (2022) apply a supervised machine learning approach to coding party manifestos of 99 parties.

Additionally, there are several projects that measure and classify parties based on expert surveys. For instance, drawing from the 2014 Chapel Hill Expert Survey, Polk et al. (2017) assess political parties' populism via the observed salience of anti-establishment and anti-elite rhetoric, as well as the emphasis displayed by parties on reducing political corruption.^3^ The PopuList project (Rooduijn et al., 2019) establishes peer reviewed unidimensional and dichotomous classification of populist, far right, far left and/or Eurosceptic parties in 30 countries.^4^ Wiesehomeier (2019) uses two waves of expert surveys to measure the degree of populism in 165 political parties and 18 presidents in 18 Latin American countries. She focuses on two dimensions: people-centrism and anti-elite morality and adopts a “bundle approach” combining different attributes in a single metric on a continuum between populist and pluralist poles of dimensions: people-centrism and moral anti-elitism. Meijers and Zaslove (2021) elaborate on the multidimensionality of the populist construct in their ambitious Populism and Political Parties Expert Survey (POPPA) conducted in 28 European countries and covering 250 political parties. Their instrument includes 16 items and captures five dimensions: Manichean worldview, indivisible people, general will, people-centrism and anti-elitism (Meijers and Zaslove, 2021, p. 385).

Even larger in scope, Norris (2020, p. 9) Global Party Survey, is presented as a departure from the ideational tradition and designed to estimate ideological values, issue positions and the degree of populist rhetoric, covering 1,052 parties in 163 countries. This expert survey asks respondents to place parties on a 11-point scale from 0 (“Strongly favors pluralist rhetoric”) to 10 (“Strongly favors populist rhetoric”),^5^ and adds other five alternative indicators aiming to capture two dimensions: (1) the only legitimate authority lies with “the people”, and (2) the critique to the corrupt, self-serving and out of touch “establishment” (Norris, 2020, p. 2–9). Similarly, the Comparative Study of Electoral Systems (2020) also offers an evaluation of political parties according to their degree of populism. Experts are given Albertazzi and McDonnell (2008, p. 3) definition of populism and requested to assign score from 0 (“not at all populist”) to 10 (“very populist”).

In sum, there are several competing approaches to measure the degree of populism of political parties. Although, most of these instruments have been developed based on similar, often ideational, definitions of populism, they diverge in several aspects. Not all studies embrace degreeism or the multidimensionality of the concept. Moreover, while most demand-side studies use large-n surveys on citizen's views as source of data, those on political parties diverge in what is considered the specific object of analysis. Some of them circumscribe their assessments to party manifestos or political communications, while others are based upon wider assessments on parties' policies, strategies or rhetoric.

## Materials and methods

This paper develops an analysis of congruence of demand- and supply-side of populism, not as an attempt to settle the theoretical debate on whether populist voters support populist parties, but as an avenue to reveal the major challenges and limitations associated to current populism measurement tools. We selected CSES Module 5 “Democracy Divided? People, Politicians and the Politics of Populism” as a dataset because it is the sole research project that incorporate data on both, the degree of populism of political parties and voters' attitudes. This way, discrepancies observed between the populist supply and demand would not be attributed to a different source of data. Additionally, CSES covers countries from several continents, which is an important feature to detect potential cross-cultural validity issues (Davidov et al., 2014).

CSES Module 5 focuses on three core dimensions: “(i) attitudes toward political elites; (ii) attitudes toward representative democracy and majority rule; (iii) attitudes toward out-groups” (Hobolt et al., 2016, p. 5–10).^6^ By following this conceptualization, they argue that the core aspect of populism is the clear distinction and antagonism between the (good) people and the (corrupt) elite (Mudde and Rovira-Kaltwasser, 2013). The political elite is accused of not acknowledging, understanding or caring about the needs people have and, consequently, not being able to deliver the public goods and services people need. In the CSES dataset question items Q4b, Q4c and Q4d measure such different aspects of negative attitudes toward the elite (Table 1).^7^

**Table 1:** Eight items to measure populist attitudes, CSES Module 5^a^.

**Items**	**Original**	**Wording of the question**	**Populist dimension**
Pop1^b^	Q4a	What people call compromise in politics is really just selling out one's principles.	*Democracy:* Challenges to representative democracy
Pop2	Q4b	Most politicians do not care about the people.	*Elite:* Attitudes toward political elites
Pop3	Q4c	Most politicians are trustworthy	*Elite:* Attitudes toward political elites
Pop4	Q4d	Politicians are the main problem in COUNTRY	*Elite:* Attitudes toward political elites
Pop5	Q4e	Having a strong leader in government is good for COUNTRY even if the leader bends the rules to get things done.	*Democracy:* Challenges to representative democracy
Pop6	Q4f	The people, and not politicians, should make our most important policy decisions.	*Democracy:* Challenges to representative democracy
Pop7	Q5a	It is better for society if minorities maintain their distinct customs and traditions.	*Out-groups:* Attitudes toward out groups
Pop8	Q6a	How important do you think the following is for being truly [NATIONALITY]. Very important, fairly important, not very important, or not important at all? a. To have been born in [COUNTRY]	*Out-groups:* Attitudes toward out groups

a Trying to be parsimonious, we just select 8 indicators for populism: 3 items to measure anti-elite; 3 items to measure democracy and 2 items to measure attitudes toward out groups. We have not included Q7, Q5b-d and Q6b-d. Additional robustness checks confirm that the findings keep in the same direction with the battery of items not included.

b Item Pop1 was not included in the survey in Ireland and Greece.

Source: Own elaboration based on Comparative Study of Electoral Systems (2020).

The perceived “institutional crisis of representation” resulting from the wrongdoings and incompetence of the elite (Rooduijn et al., 2014) is the second populist dimension captured in the CSES study. Populists usually propose to overcome the problem of representation either by empowering a strong charismatic leader who would embody and voice the will of the people (Müller, 2016, p. 32–38), or by involving more directly “the people” in direct decision making (Mohrenberg et al., 2021). Compromise also clashes with the antagonistic and Manichean view of politics dynamics and as such is perceived as a sort of betrayal to the interest of the people. These ideas on the populist conception of democracy are captured by items Q4a, Q4e and Q4f in the CSES dataset (Table 1).

Finally, the CSES Module 5 offers additional survey items related to attitudes toward out-groups. In-group homogeneity and exclusion are also key elements of populists' interpretation of society (Jagers and Walgrave, 2007, p. 323; Pellegrini, 2022). The underserving and corrupt minorities—e.g., “the elite”, “the colonizers”, “the immigrants”—, do not really belong to the *demos* or the “heartland” (Taggart, 2000), the “true people” must fight to “have their country back” (Panizza, 2017, p. 409–411). Items Q5a and Q6a in the CSES dataset reflect these ideas (Table 1).^8^ Regarding the assessment of the supply-side of populism, CSES Module 5's country experts are presented with a definition and asked to assess on a 11-point scale the degree of populism of each political party.^9^ This assessment was made by a variable number of country experts, who may or not be specialist on the area of populism, ranging from 1 (Australia, Hungary and Italy country reports) to 43 experts (Greece report) (Table 2).

**Table 2:** List of parties classified as populist (score >5)^a^.

**Country (election year)**	**Populist parties Comparative Study of Electoral Systems (2020)**	**Populist parties Meijers and Zaslove (2020)^b^**
Australia (2019)	One Nation (7) United Australia (8) (based on the assessment of 1 country expert)	
Austria (2017)	Austrian People's Party [ÖVP] (6) Freedom party of Austria [FPÖ] (9) Liste Peter Pilz (6) (based on 11 country experts)	Freedom party of Austria [FPÖ] (8.89)
Brazil (2018)	AVANTE—“Go forward” (6) DC—Christian Democracy (6) DEM—Democrats (6) MDB—Brazilian Democratic Movement (7) PATRI/Patriota- Patriot (7) PCdoB—Communist Party of Brazil (8) PDT—Democratic Labor Party (8) PODE/Podemos—“We can” (7) PP—Progressive Party (6) PPS—Popular Socialist Party (6) PR—Republican Party (7) PRB—Brazilian Republican Party (7) PROS—Republican Party of Social Order (6) PRP—Progressive Republican Party (7) PSC—Christian Social Party (6) PSD—Social Democratic Party (6) PSL—Liberal Social Party (10) PT—Workers' Party (10) PTB—Brazilian Labor Party (8) SD—Solidarity (7) (based on 3 country experts)	
France (2017)	La Republique en Marche (6) France Insoumise (8) Front National [FN] (10) (based on 3 country experts)	Front National [FN] (9.07) France Insoumise (8.44) France Arise (DLF) (7.42) New Anticapitalistic Party [NPA] (7.75) Parti Communist (8.71)
Germany (2017)	Alternative for Germany [AfD] (7) (based on 3 country experts)	Alternative for Germany [AfD] (9.44)
Greece (2015)	Synaspismos tis Rizospatikis Aristeras [SYRIZA] (8) Laikos Syndesmos - Chrisi Avgi (Golden Dawn) [LS- XA] (8) Kommounistiko Komma Ellados [KKE] (8) Anexartitoi Ellines [ANEL] (7) Enosi Kentroon [EK] (6) (based on 43 country experts)	Synaspismos tis Rizospatikis Aristeras [SYRIZA] (7.63) Laikos Syndesmos - Chrisi Avgi (Golden Dawn) [LS-XA] (9.12) Kommounistiko Komma Ellados [KKE] (7.51) Anexartitoi Ellines [ANEL] (8.46) Enosi Kentroon [EK] (6.3) Popular Unity [LAE] (8.9)
Hong Kong (2016)	Democratic Alliance for Betterment and Progress of Hong Kong (6) Hong Kong Federation of Trade Unions (8) Youngspiration (8) (based on 2 country experts)	
Hungary (2018)	FIDESZ—Hungarian Civic Alliance (9) Christian Democratic People's Party [KDNP] (9) Hungarian Socialist Party [MSZP] (6) Movement for a Better Hungary [JOBBIK] (7) (based on 1 country expert)	FIDESZ—Hungarian Civic Alliance (9.01) Movement for a Better Hungary [JOBBIK] (7.3)
Iceland (2017)	Center Party (6) People's Party (6) (based on 3 country experts)	
Ireland (2017)	Sinn Féin [SF] (6) United Left Alliance (7) (based on 2 country experts)	Sinn Féin [SF] (6.23)
Italy (2018)	Movimento 5 Stelle/5 Star Movement [M5S] (10) Lega/League (9) Fratelli d'Italia/Brothers of Italy [FdI] (7) (based on 1 country expert)	Movimento 5 Stelle / 5 Star Movement [M5S] (9.45) Lega/League (8.60) Forza Italia [FI] (5.56) Fratelli d'Italia/Brothers of Italy [FdI] (7.44)
Lithuania (2016)	Anti-corruption Coalition of N. Puteikis and K. Krivickas (9) Lithuanian Polish Electoral Action—League of Christian Families (6) Order and Justice Party [TT] (8) (based on 3 country experts)	Order and Justice Party [TT] (7.07)
Montenegro (2016)	Democratic front (8) DEMOS (6) Socialist People Party (6) Democrats (6) (based on 6 country experts)	
Norway (2017)	The Progress Party (7) (based on 5 country experts)	
New Zeeland (2017)	Labor (6) National (7) New Zealand First (9) ACT (7) Mana (6) (based on 1 country expert)	
South Korea (2016)	People's Party (7) (based on 5 country experts)	
United States (2016)	Republican Party (8) Libertarian Party (9) (based on 2 country experts)	

a In brackets the punctuations of populism from 0 “not at all populist” to 10 “very populist”.

b Meijers and Zaslove (2020) only measure European countries. Their survey was fielded between April 2018 and July 2018. CSES assessments took place between 2016 and 2019, depending on the country.

Source: own elaboration base on CSES Module 5, 14 May 2020 update and Meijers and Zaslove (2020).

The documentation provided^10^ does not seem to indicate consistency checks across cases. There is at least one country, Chile, containing some apparent mistakes and two countries, Taiwan and Turkey, on which no score on populism was provided by the country experts.^11^ Given some of the abovementioned limitations, we include Meijers and Zaslove's (2020) POPPA measurement as a robustness check in the analysis of congruence between the CSES classification of political parties and the CSES data on populist attitudes. Table 2 shows the parties that were considered populist, i.e., those that received a score of more than 5 on the 11 scale in the CSES and POPPA datasets.^12^

As previously mentioned, we assess the extent to which populist attitudes indicators (Table 1) correlate with the higher tendency of voters to support parties that received a populist score. We examine eleven European countries and six non-European ones: Australia, Austria, Brazil, France, Germany, Greece, Hong Kong, Hungary, Iceland, Italy, Ireland, South Korea, Lithuania, Montenegro, New Zealand, South Korea, and the United States of America.^13^ The sample contains between 23.000 and 29.000 individuals.

We run specific logistic regressions in each country to measure the relationship between each of the eight items capturing populist attitudes in the CSES survey (Table 1) and the likelihood to vote for a populist party. Populist items (pop1, pop2, etc.) are included individually, alongside some control variables (see below), to avoid problems of collinearity. We make the original continuous classification dichotomous (1 = populist party; 0 = non-populist party), considering as non-populist those parties with a score of 5 or less and populist those with scores from 6 to 10.^14^ Table 3 displays the expected effect of each item on the dependent variable—i.e., support for populist parties.^15^

**Table 3:** Expected effect of populist dimensions upon the vote for populist parties.

**Items**	**Populist dimension**	**Wording of the question**	**Possible values**	**Expected effect**
pop1	*Democracy*	What people call compromise in politics is really just selling out one's principles.	(1 = strongly agree−5 = strongly disagree)	Negative
pop2	*Elite*	Most politicians do not care about the people.	(1 = strongly agree−5 = strongly disagree)	Negative
pop3	*Elite*	Most politicians are trustworthy	(1 = strongly agree−5 = strongly disagree)	Positive
pop4	*Elite*	Politicians are the main problem in COUNTRY	(1 = strongly agree−5 = strongly disagree)	Negative
pop5	*Democracy*	Having a strong leader in government is good for COUNTRY even if the leader bends the rules to get things done.	(1 = strongly agree−5 = strongly disagree)	Negative
pop6	*Democracy*	The people, and not politicians, should make our most important policy decisions.	(1 = strongly agree−5 = strongly disagree)	Negative
pop7	*Out-groups*	It is better for society if minorities maintain their distinct customs and traditions.	(1 = strongly agree−5 = strongly disagree)	Negative
pop8	*Out-groups*	How important do you think the following is for being truly [NATIONALITY]… very important, fairly important, not very important, or not important at all? a. To have been born in [COUNTRY]	(1 = very important−4 = not important at all)	Negative

Source: Own elaboration based on CSES Module 5.

For the sake of robustness, and using the original (continuous) scores of populism given by the experts to each party, we replicate this analysis using ordinary least squares (OLS) regressions and find results consistent with those in our logistic model (Figures A1, A2 in the Appendix). All countries display equivalent results in both logistics and linear regressions. France is absent from our OLS model, due to some missing values for non-populist parties. We also run logistic and linear regressions for the pooled dataset specifying country fixed effects (see Table A1). The results are consistent with those in the abovementioned country by country analyses.

To explain some inconsistencies observed between the supply- and demand- side of populism, we carry out additional analysis changing the nature of our independent variables, turning the eight populist items into an additive index. For the political parties classified by the CSES as populists and with a significant number of voters (N ≥ 70) we conduct logistic regressions, to ensure that mismatch between populist attitudes and vote for populist parties is, as well, party-specific (see Figures A5, A6).

Table 4 describes the variables considered in the logistic models. We include three controls (sociodemographic variables) in the models: *Gender* (1 = male); *Year of birth* (continuous variable) and *Education level* (1 = University degree). The last column shows the variance inflation factors (VIFs) of all variables. All VIFs are well below the levels that would rise concerns of collinearity (James et al., 2017, p. 59–120); the mean VIF is 1.24 and the maximum one 1.71 (pop1).^16^

**Table 4:** Description of variables.

**Variable**	**Observations**	**Mean**	**Standard deviation**	**Min**.	**Max**.	**VIF**
Gender	29.044	0.51	0.50	0	1	1.01
Year of birth	28.901	1968	17.41	1,916	2,002	1.08
Education	26.618	1.14	0.68	0	2	1.15
pop1	23.397	2.93	1.24	1	5	1.23
pop2	28.630	2.64	1.30	1	5	1.71
pop3	28.549	3.43	1.21	1	5	1.35
pop4	28.299	2.90	1.31	1	5	1.67
pop5	28.151	3.19	1.38	1	5	1.14
pop6	28.429	2.60	1.28	1	5	1.28
pop7	28.472	2.26	1.20	1	5	1.14
pop8	28.099	2.39	1.07	1	4	1.19

Source: Own elaboration based on CSES Module 5.

## Results

**Figure 1:**
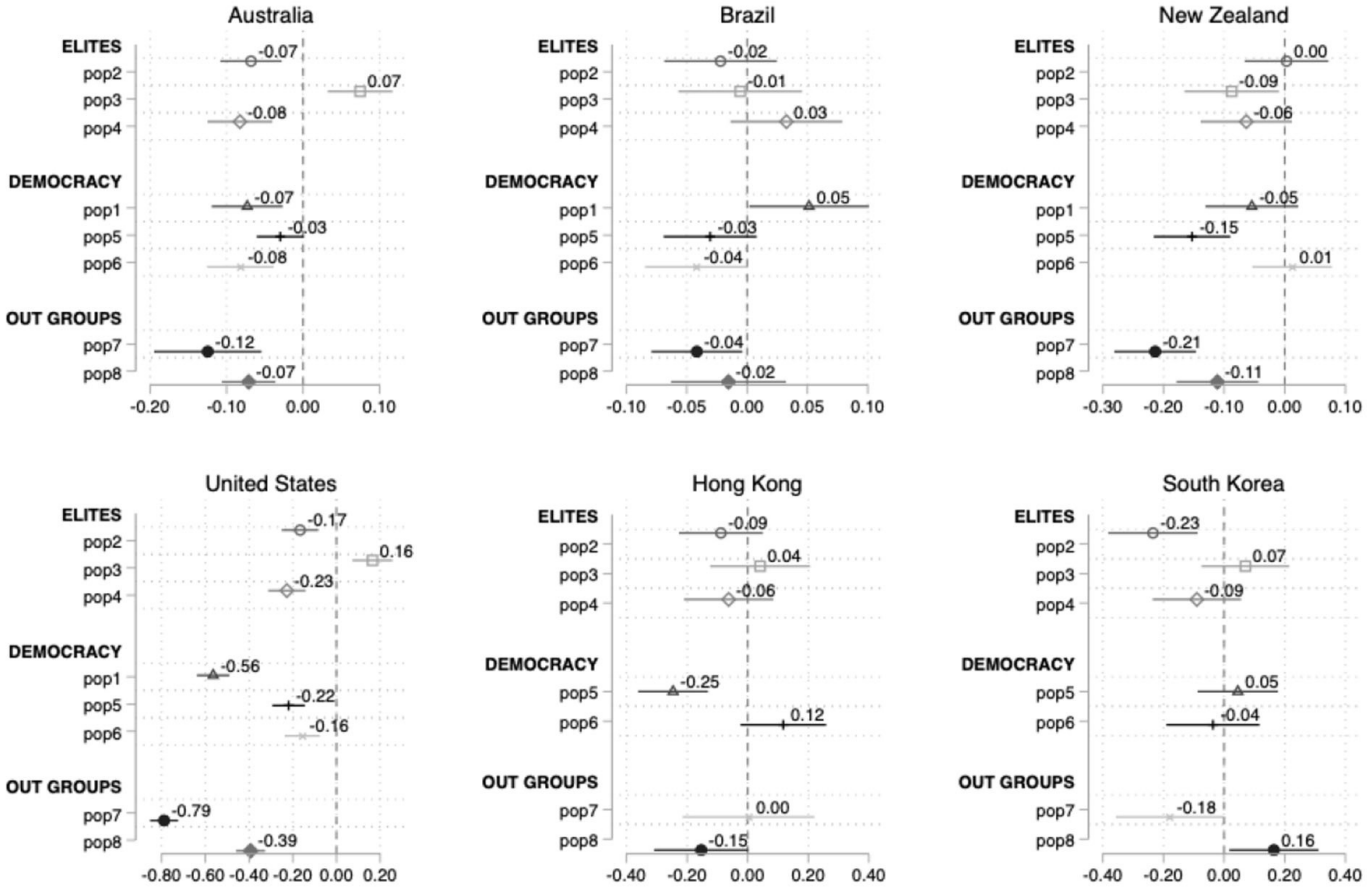
Average marginal effects on the probability to cast a vote for a populist party, non-European countries.

**Figure 2:**
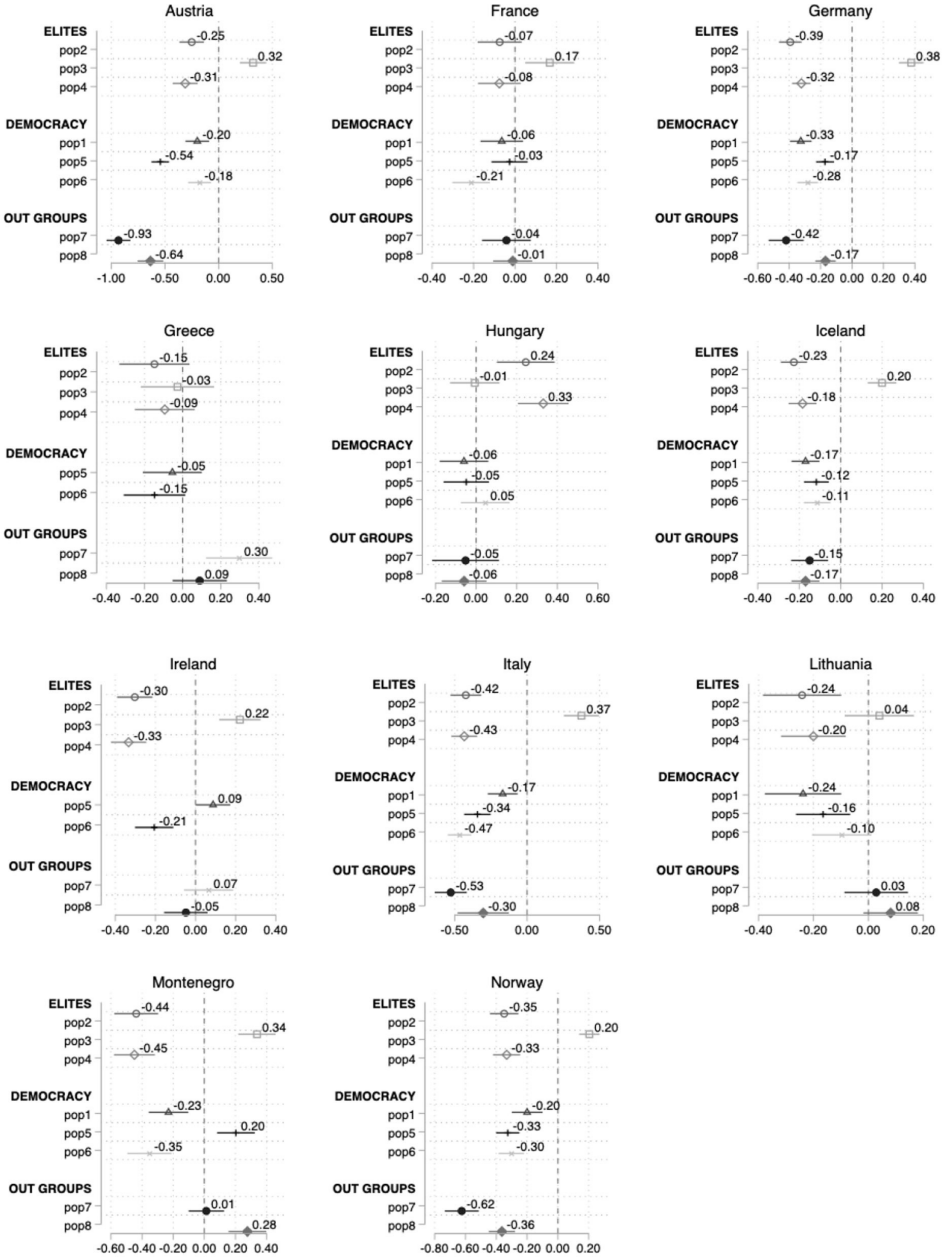
Average marginal effects on the probability to cast a vote for a populist party, European countries.

Our analysis indicates that, although there is a significant statistical correlation between populist attitudes and support for parties classified as populist in the USA, Australia and most European countries, there are still many other countries—including, Brazil, New Zealand, South Korea, Hong Kong, Hungary, France, and Greece—where we fail to observe congruence between CSES Module 5 supply and demand measurements of populism. Figures 1, 2 show the impact of the abovementioned eight attitudinal items, controlling by the sociodemographic factors (not shown), upon the probability to cast a vote for a populist party in six non-European (Figure 1) and eleven European countries (Figure 2).

One limitation of the logistic regression coefficients is that they do not provide information regarding the comparative magnitude of each covariate's effect. Therefore, after running the logistic regression we calculate their average marginal effects (AMEs) that capture the average changes in the probability of vote from a populist party instead of a non-populist party.^17^ The AMEs are calculated as follows: for each observation of the dataset, the marginal effect of a given variable on our dependent variable is estimated (holding all other independent variables constant), and then these estimations are averaged for all the observations (Williams, 2012). Each horizontal line in Figure 1 represents an independent variable of the model, the point standing for the best estimation of its effect upon the dependent variable, and the horizontal line covering the 95% confidence interval. If a confidence interval crosses the vertical line drawn at the zero value of the horizontal axis (representing the absence of effects), the effect of the variable is not statistically significant. If it does not and is located to its right, the effect is positive and statistically significant; whereas if it is located to its left, the effect is negative and statistically significant.

Figure 1 clusters the eight items into three big blocks: negative attitudes toward elites (*elite*); democratic values (*democracy*) and out groups considerations (*out-groups*), following the three dimensions specified by Hobolt et al. (2016). In the case of Australia, except for pop5 (strong leader), items help to understand populist parties support, being pop7 (minority rights) the one with the higher coefficient (−0.12). Brazil clearly means a case where the populist attitudes items do not seem correlated with support for this set of surmised populist political forces. Most of the items are non-statistically significant (they touch the vertical line). Regarding New Zealand, it is surprising that, while both of the items that measure the *out-groups* dimension are statistically significant and display high effects, the cluster of *democracy* and *elite* do not have any effect upon the likelihood to vote for a populist force. The cases of Hong Kong and South Korea goes in the same line than the Brazilian one, i.e., the populist attitudes items do not seem correlated with a higher probability to vote for parties presumed populist. USA is the non-European country with the best fit between CSES measurements of supply and demand-sides of populism, as all items help to understand the vote for populist leaders (the Republican Party and the Libertarian Party). Items pop7 and pop8 (both belonged to the *out groups* dimension), with −0.79 and −0.39 coefficients respectively, and pop1 (*democracy*) (−0.56) are particularly helpful in explaining support for populist parties.

Figure 2 shows a greater explanatory capacity of the eight attitudinal items in the case of most of the European countries analyzed, even controlling for sociodemographic variables. However, we still observe that in some countries—i.e., France, Hungary, Lithuania, Montenegro, Ireland, and Greece—there is no significant correlation between some of the populism items and preference for populist parties. In Austria, Germany, Iceland, Italy, and Norway the battery of items employed to measure populist attitudes works well as predictors. Items such as pop5 (strong leader) and pop7 (minority rights) exhibit high coefficients (−0.54, and −0.93, respectively). Similarly, in Germany *elite* items pop2 and pop3 and *out-groups* item pop7 display the strongest effect.

Despite the ideological discrepancies among the Italian parties ranked as populist at the CSES Module 5 (M5S, *Lega* and Brothers of Italy), all the attitudinal items are statistically significant, follow the expected direction and exhibit high coefficients (specially pop7, pop6, pop4, and pop2). Norway and Iceland demand- and the supply-side demand as also congruent. It is worth noting that in Norway, pop7 (minority rights) obtains a relatively very high coefficient (−0.62), only lower than the one in Austria (−0.93) and the USA (−0.79). This is probably logical given that the only party considered populist in that country is the Progress Party, a populist radical right force with a notorious anti-immigration agenda. Pop7 is precisely the only item that is not significantly correlated with support to populist parties in Montenegro, although in this country pop5 displays an effect opposite to the one we could theoretically expect.

Conversely, the capacity of these items to predict support for parties classified as populist is more limited or inexistent in other European cases. Only four items are statistically significant in the cases of Ireland (pop2, pop3, pop4, and pop6) and Lithuania (pop1, pop2, pop4, and pop5), none of which belong to the *out-groups* dimension. Only 2 items show a significant association in the cases of France (pop3, and pop6) and Hungary (pop2, and pop4) but in the latter items have an effect opposite the one expected. Finally, in Greece, the single item with statistically significant impact (pop7) also goes in the opposite sense to the intention of the designers of the instrument.

**Figure 3:**
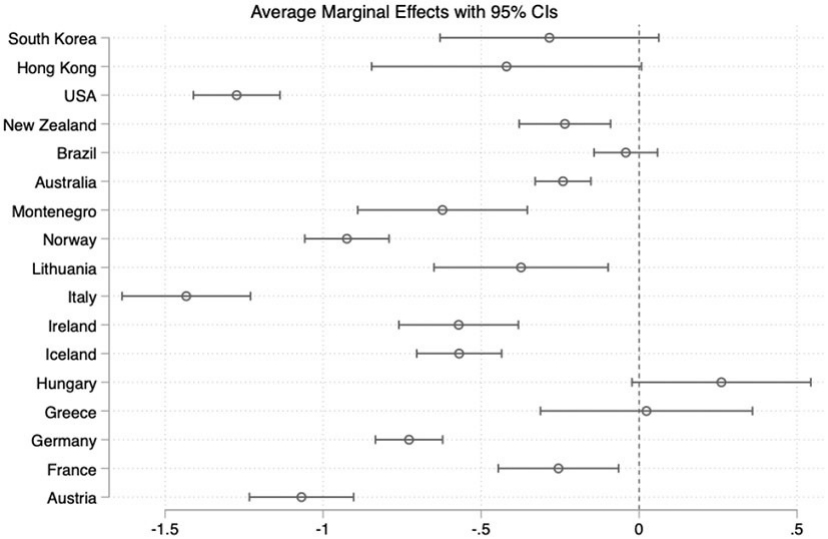
Average marginal effects on the probability to cast a vote for a populist party instead of non-populist parties (all countries in populist index).

Given the apparent limited ability of the CSES items to capture voters prone to support populist parties when considered individually, and following the footsteps of Akkerman et al. (2014) and Elchardus and Spruyt (2016), we construct an additive index with the eight populism items to check whether at least the combined presence of these populist beliefs serves as a predictor of support for populist parties.^18^ Figure 3 lays out the relationship between the unidimensional populist index and the probability to vote for a populist party country by country. Figures A3, A4 in the Appendix provide a different graphical approximation, i.e., a margins plot with the linear prediction of support for populist parties—continuous variable—based on the populist attitudes index. Yet, the additive index models do not show significant improvement vis-à-vis the previous itemized models. There are still 5 countries out of 17 with no statistically significant relation between populist attitudes and support for populist parties, two of which (Hungary and Greece) exhibit a negative coefficient.

The inability of the attitudinal items to discriminate “populist” from “non populist citizens” can be one of the sources of the mismatch between supply- and demand-side measurements. Next section explores three potential causes for the congruence issues encountered in the analysis of the CSES Module 5 dataset: (i) problems with the design of items in attitudes surveys; (ii) problems with the experts' scores and classification of parties; (iii) some dimensions or attributes considered in supply and demand-side measurements may be specific to certain types of populism.

## Discussion: What fails? Potential sources of incongruence between the supply- and the demand- side measurement instruments

### Choice and design of attitudinal items

What if the observed incongruence is caused by the choice or design of attitudinal items? Although most scales rely on an ideational approach, there is not a widespread agreement on which are “the best” items and, in fact, we find very limited overlap across scales (Castanho Silva et al., 2020). There is always a risk that items do not properly capture the populist dimensions/attributes intended. Additionally, even if they prove effective in some case studies, certain wordings of items/questions do not travel well and elicit different interpretations in other country contexts. This may be the reason why some scales yield some surprisingly disparate results in different countries.^19^

**Figure 4:**
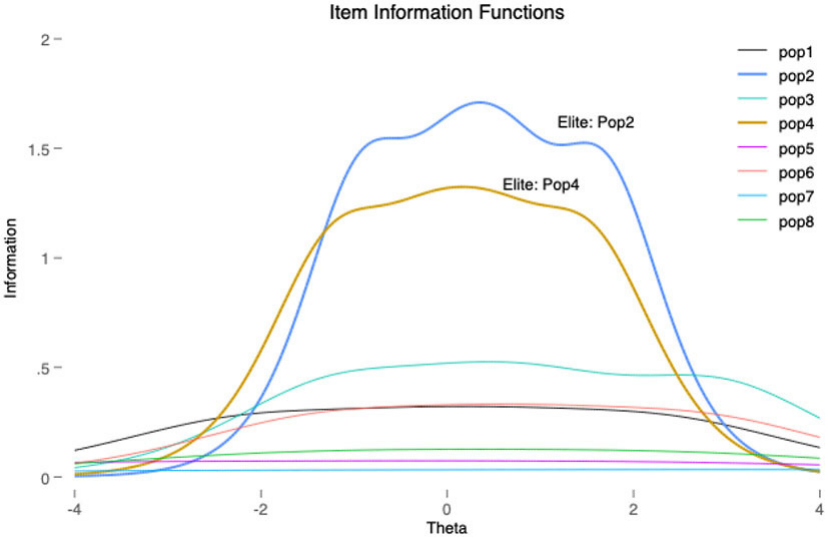
Individual item characteristic curves.

To prevent these problems, some scales are refined during their development by applying confirmatory factor analysis (CFA) (Akkerman et al., 2014; Castanho Silva et al., 2018; Schulz et al., 2018) or item response theory (IRT) (Van Hauwaert and van Kessel, 2018; Van Hauwaert et al., 2020) as means to eliminate items that do not load sufficiently on the desired latent dimension.^20^ In order to assess the impact of each CSES Module 5 item upon the probability to support a populist party, and given their continuous nature, we use a graded response model (GRM)—that is an IRT model typically used in health and psychology related surveys (Depaoli et al., 2018)—to analyse the relative fitness of each of the items in the CSES Module 5 instrument (see Figure 4).^21^

Our preliminary analysis of the eight items mentioned above suggests that the CSES Module 5 attitudes scale could benefit from further development or refinement. Out of the three dimensions that were meant to be captured (Hobolt et al., 2016, p. 5) only the items ascribed to the *elite* dimension (pop2, and pop4) display significant discriminatory power. The rest of the items—related to *democracy* and *out-groups* dimensions—display somewhat flat information slopes and, therefore, could probably be either excluded as redundant, rephrased, or replaced.^22^ This seems to suggest that this scale would benefit from further work of redesign, test, and validation of items. Further research is required to discern whether the problems encountered in some of the items, are associated to an unclear wording, contextual peculiarities, or deepest theoretical considerations. In any case, this illustrates that the inability of some attitudinal items to discriminate “populist” from “non populist citizens” can be one of the sources of mismatch between supply and demand-side measurements.

We run a confirmatory factor analysis (CFA) model and find only two factors with eigenvalues over one (2.56 and 1.18) and that, in line with our previous GRM analysis, pop2 displays the lowest unique variance (Table A2). Our analysis corroborates those by Castanho Silva et al. (2020) and Jungkunz et al. (2021) that also reveal important limitations regarding the choice of items by the CSES and other demand-side populism scales. The issues detected regarding goodness of fit, and unexpected loadings, indicate that at the failures predicting support for populist parties in some countries may be associated with the design of the scale. Accordingly, we can posit that CSES, and other scales, may not be properly measuring what they are supposed to measure.

It is worth considering that, as Wuttke et al. (2020) demonstrate, the operationalization strategies—i.e., Sartorian, Bollen or Goertzian—applied for the construction of composite indexes of populist attitudes, such as those used in Akkerman et al. (2014), Schulz et al. (2018), and Castanho Silva et al. (2018), may entail significant variations in terms of scores and therefore could also potentially affect the level of congruence with supply-side measurements. Following Wuttke et al.'s (2020) suggestion, Castanho Silva and Wratil (2021) and Silva et al. (2022) successfully apply non-compensatory approaches to the treatment of populist attitudinal that were part of extant scales, such as those by Akkerman et al. (2014) and Van Hauwaert and van Kessel (2018). Moreover, Kefford et al. (2021) show that some dimensions of populism, such as attitudes toward populist discursive and performative styles, traditionally absent from demand-side studies, can also be incorporated in attitudinal surveys. Similarly, recent studies demonstrate that including in the analysis different but related constructs such as narcissism (Arias-Maldonado et al., forthcoming; Mayer et al., 2020) or belief in conspiracy theories (Castanho Silva et al., 2017; Salvati et al., 2022).

Finally, Olivas Osuna (2021) goes a step further, suggesting the introduction of “intersection” items in surveys to better capture the overlapping nature of different of the theoretical attributes of populism. Drawing from the concept of “intersectionality” in gender studies (Crenshaw, 1989; McCall, 2005), he argues that intersections are worth analysing as separate variables because they may not necessarily follow an additive logic and populism may be more than the sum of its parts (Olivas Osuna, 2021, p. 846–847). Olivas Osuna (2021) also questions the widespread assumption, at least among the ideational school, that the study of populism requires to follow a classical categorization (Sartori, 1970, p. 1038) based on minimal definitions and a set of necessary attributes. In line with Wittgenstein's “family resemblances” and Lakoff's “radial structure” approaches (Lakoff, 1987, p. 16–20, 83–84; Collier and Mahon, 1993, p. 848–850), he argues that a more ambitious multidimensional stance in data gathering, would enable us to better analyse borderline cases and identify varieties within populism (Olivas Osuna, 2021, p. 832–836).

In sum, as the analysis of CSES results illustrates, several of the items that are customarily used in social research to assess populist attitudes and beliefs, may not be optimal. Recent research suggests that the theoretical assumptions concerning the populism concept structure and the choice of operationalization techniques in multidimensional scales should be carefully revisited.

### Problematic assessment of parties

Lack of congruence found in several cases in the CSES dataset between the demand and supply in the populist marketplace could also be explained by problems in the methods used to assess the degree of populism and classify parties as populists or non-populists. The use of different criteria and definitions certainly has an impact on the score received by each party. Table 2 in the Data and Methods section shows the discrepancies between the CSES Module 5 and Meijers and Zaslove (2020) POPPA classifications. Meijers and Zaslove (2021) assessment is based on 294 responses from country-experts, selected on the basis of publications records, in 28 European countries. Unlike in the case of CSES, POPPA experts were not explicitly asked to assess populism, but a set of dimensions associated to this phenomenon in line with the ideational approach (Meijers and Zaslove, 2021, p. 382–385).

Although some parties receive similar scores in both scales, we find significant differences in others. Some of them score much lower in the POPPA populism measurement, such as the Hungarian Socialist Party MSZP (−3.55 points difference), Austrian *Liste Peter Pilz* (−3.03 points difference), ÖVP (−2.2 points difference), and French *La République en Marche* (−1.57 points difference). On the other hand, some parties obtain a significantly higher score in the POPPA classification—i.e., the German AfD (2.44 points difference), Italian *Forza Italia* (1.56 difference) and the Greek ANEL (1.43 points difference). The French Communist Party, which according to Meijers and Zaslove (2020) classification scores very high, was not assessed in the CSES expert survey.

**Figure 5:**
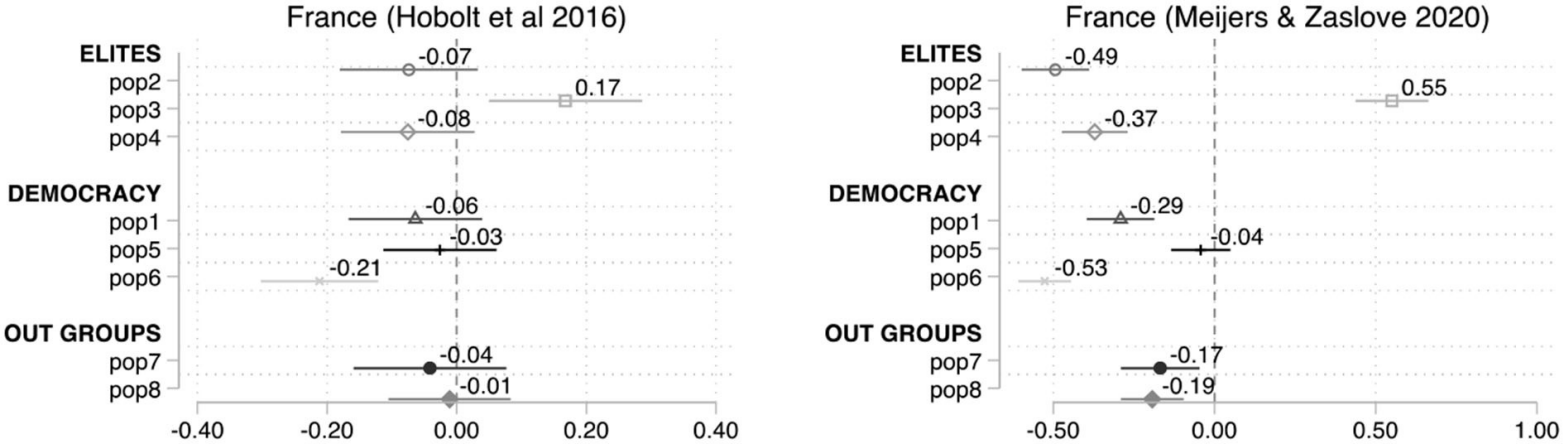
Average marginal effects on the probability to cast a vote for a populist party instead of other parties, the case of France.

**Figure 6:**
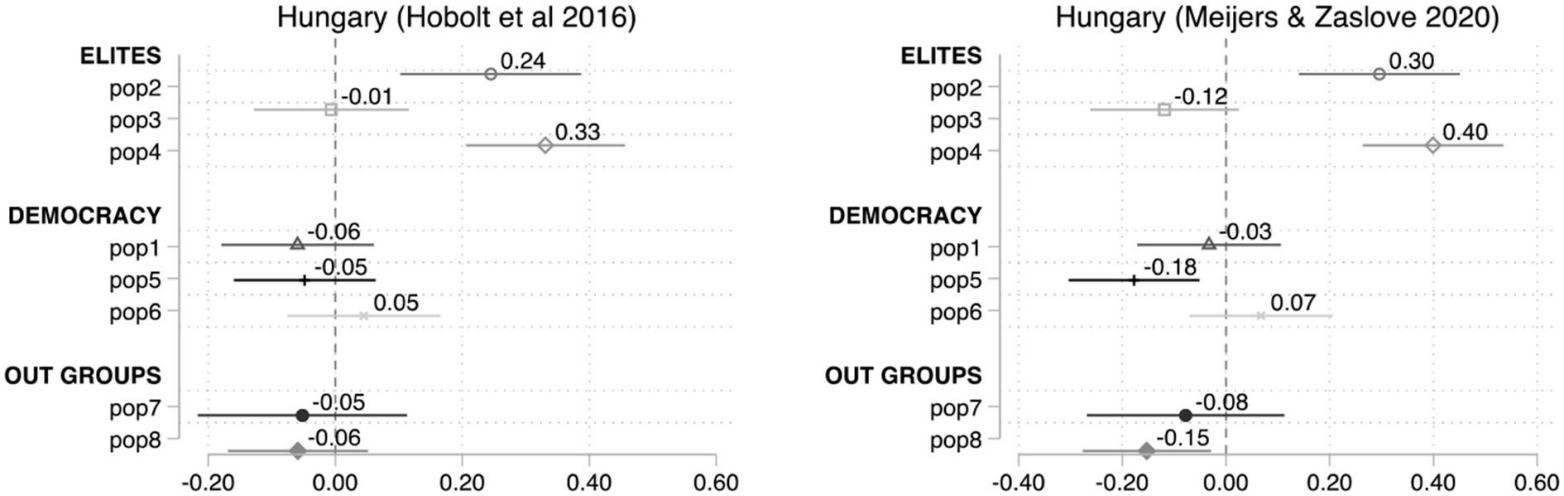
Average marginal effects on the probability to cast a vote for a populist party instead of other parties, the case of Hungary.

Figures 5, 6, replicate the analysis for France and Hungary—both displaying particularly poor congruence—but using Meijers and Zaslove (2020) POPPA scores (right panels) to estimate the dependent variable (Table 2). The result for the French case show a clear improvement in the level of congruence. With just the exception of pop5, the rest of the items become statistically significant and go in the expected direction when using POPPA classification, which excludes *La République en Marche* and includes the French Communist Party. Furthermore, some of the other issues exhibit higher coefficients—i.e., pop6 (−0.53), pop2 (−0.49), and pop4 (−0.37). The relatively good fit of the CSES attitudinal items with the POPPA supply-side classification seems to indicate that in the case of France the problem of congruence is not so much due to the choice of attitudinal items but to the assessment made by the CSES experts on the level of populism of the parties included.

CSES acknowledges the multidimensionality of the phenomenon, and accordingly designs items to capture some of these components in citizens' attitudes (Hobolt et al., 2016, p. 5–10). Nevertheless, this logic was not followed when assessing parties, and CSES experts were not requested or had the choice to reflect on different components or attributes. CSES Module 5 simply provides a definition but does not require a justification on why the overall score was achieve. More explicit rules in terms of the criteria or dimensions that should be taken into consideration or potential benchmarks which could be considered in the evaluation may help standardize the analysis across experts and countries. More recent studies, such as Meijers and Zaslove (2020; 2021) POPPA and Norris (2020, p. 10) Global Party Survey, on the other hand, include expert survey items which try to mirror those usually encountered in the demand-side scales, and conduct robustness tests and comparisons with other measures.^23^

However, Figure 6 shows problems of congruence between the supply- and demand-side measurements in Hungary even when adopting the POPPA classification. Half of the items (pop3, pop1, pop6, and pop7) are not correlated in a statistically significant way with support to populist parties. Interestingly, another two items, pop2 and pop4, being statistically significant, display unexpected directions (positive). Thus, the Hungarian example indicates that the mismatch cannot be exclusively attributed to party classification.

Country-specific cultural and political characteristics deserve further attention as voters may not understand the questions in the same way in every country and in some of them, parties classified as populist may not differ so significantly from their competitors (Pirro, 2015). Although the CSES used a gradient system and did not include any explicit threshold to distinguish “populist” from “non populist” parties, some of the existing studies on the supply-side include binary (Rooduijn et al., 2019) or three-ways (Hawkins, 2009) assessments. These approaches often lead to additional problems especially in borderline cases or in studies where the classification of parties as “populist” serve as basis or filter to study other aspects of populism, such as electoral behavior or Eurosceptic views. For instance, applying the PopuList classification, Taggart and Pirro (2021, p. 285–288, 291) show that cumulative populist party vote share in 2019 national elections in a comparative analysis of 30 European countries ranges from 66.6% in Italy and 62.2% in Hungary, to 3.2% in the UK and 1.5% in Portugal.^24^ This illustrates how a classificatory approach to the study of populist-supply can obfuscate our understanding of a much more complex reality. Although in the UK and Portugal, only a small set of parties are formally classified as populist, we cannot conclude that populist attitudes and populist voters are negligeable in these countries. Studies investigating the supply-side of populism should be aware of these shortcomings and consider multidimensional gradient approaches that could mirror the research currently conducted on the demand-side. The following subsection expands on how the degree of congruence may be also impacted by the specific type of party.

### Conceptualization biases

Differences in the conceptualization—or emphasis on specific populist attributes—can also explain why some approaches work in some regional contexts but not so much in others (De la Torre and Mazzoleni, 2019, p. 81–85, 90–91). For instance, the emphasis on the anti-elite dimension can hinder the ability of some measurements to properly assess populism in countries where populist parties are in government (Jungkunz et al., 2021). Similarly, the conflation of populism with exclusionary right-wing nationalism in the literature (De Cleen and Stavrakakis, 2017) contributes to create some problems when it comes to measure attitudes and parties. The existence of varieties within populism (Berlin, 1968, p. 138–155; Müller, 2016, p. 11–19) makes more difficult the process of selecting items and components which should be wide enough to encompass different types of movements but at the same sufficiently specific to discriminate populist from non-populist profiles on both sides of the left-right continuum.

Our analysis at a party level confirms that the capacity of the items in the CSES scale to predict vote for specific parties varies widely. Figures A5, A6 in the Appendix, show that CSES items in general fails to detect supporters for many of left-leaning parties with high populist scores—the results are particularly poor for *La France Insoumise*, SYRIZA and *Partido dos Trabalhadores—*. Although CSES items are better predictors of support for right-wing populist parties, there are also some exceptions. Only pop7 seems to be correlated with support for *Partido Social Liberal* and *Lega*, and in the case of *Fidesz* we find that pop1 and pop8 show no significant correlation and pop7 a relationship which is opposite to the expected one.^25^

**Figure 7:**
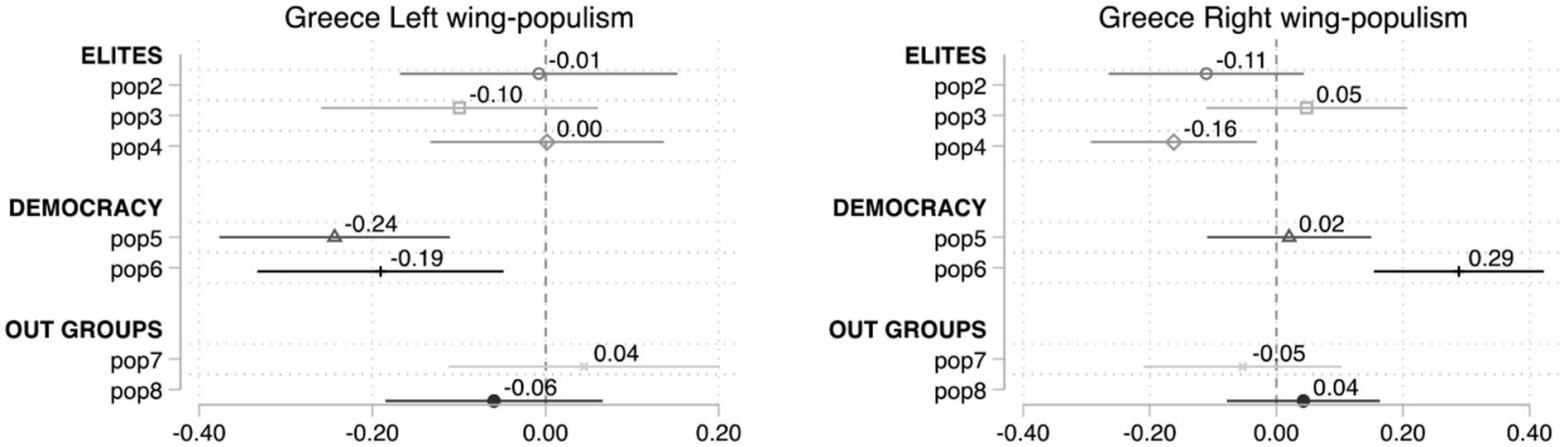
Average marginal effects on the probability to cast a vote for a populist (left *vs* right) party, Greece.

The case of Greece helps to further illustrate this issue. Figure 7 disentangles left- and right-wing Greek populist parties and reveals several interesting findings.^26^ Firstly, pop4 (*elite* dimension) works as a better predictor for the right-wing populist parties than for the left-wing ones. Secondly, pop7 (*out-groups* dimension), which is the sole statistically significant item when considering all populist parties together, cease to become a statistically significant predictor in both, the case of right and left-wing populism. This contradicts the generally held assumption that left-wing populism is predominantly in favor of allowing minorities to maintain their distinct customs and traditions. Yet this is not completely surprising as previous research suggests that left-wing populist parties may sometimes display a nativist component (Santana and Rama, 2018). Finally, pop6, although did not have any statistically significant impact when considering all populist parties together, displays opposite, and significant, results in left- and right-wing populism. This suggests that, at least in Greece, voters of left-wing populist parties are statistically in favor of more direct democracy, while supporters of the right-wing populist parties prefer to keep the decisions politicians' hands.^27^

The disparate performances of CSES Module 5 items observed in our analysis resonate with the results of other recent studies that use different scales. Marcos-Marne (2021) shows that populist attitudes, as reflected in the Akkerman et al. (2014) scale, tend to activate vote for left-wing parties more than for right-wing parties. Silva et al. (2022) found that populist attitudinal items have a very small role in explaining Bolsonaro's success in Brazil's 2018 presidential elections. Using as reference Schulz et al. (2018) scale, Hameleers et al. (2021) prove *via* an experiment conducted in 15 European countries, that different messages and frames can activate different attitudinal dimensions of populism—such as anti-elitism, belief in a homogenous people or support for popular sovereignty—independently from each other, but that the effects of populist messages largely depend on the characteristics of countries and level of exposure to the messages. Finally, Kefford et al. (2021) show that in the Australian case, attitudes toward populist communication—emerging from the discursive-performative tradition—display a strong effect on populist right voting, independent from that of the ideational items that have thus far monopolized most scales.

In sum, it may be worth accepting that no minimal definition, brief attitudes scale, or classificatory scheme may prove adequate for capturing the diversity encountered in the populist marketplace, across countries and ideological divides. Some of the existing items and criteria work well only when considering a specific type of populism, or in certain geographical areas which indicates that it may be time to reconsider and expand our repertoire.

## Conclusions

Numerous sociological studies have focused on how to measure populist attitudes and beliefs *via* survey analysis. Similarly, social scientists have devised several tools to classify political parties as populist and non-populist. The study of the congruence between the demand- and supply-side of populism is key to better understand different dynamics and ideational links between populist parties and their potential voters. Unfortunately, these studies remain scarce and most existing measurement instruments were not created paying much consideration to their compatibility with those applied in the other side of this supply-demand divide. Our analysis critically engages with the state of social scientific research in this area and identifies a set of problems that most studies in the field are not sufficiently addressing.

Although the analyses of demand- and supply-side of populism have grown in popularity and sophistication, they have followed parallel but separate ways. Furthermore, there are still open conceptual debates about what definitional attributes of populism we should prioritize when assessing populist attitudes and parties. Our assessment of the methodology applied by the Comparative Study of Electoral Systems (CSES) Module 5 to capture the supply- and demand-side shows that the predictive capacity of some of the items, typically considered as indicators of populism, is limited. This article uses the influential and rich CSES study to illustrate important shortcomings and challenges that should be considered when attempting to measure populism. We acknowledge that CSES, and several other important contributions mentioned throughout this article (e.g., Hawkins, 2009; Akkerman et al., 2014; Schulz et al., 2018; Rooduijn et al., 2019), have played a key role in the development of the first few tools to measure and compare systematically this complex latent construct. They remain valuable techniques to proxy and compare populism across countries in a cost-effective manner. However, we suggest the need to recalibrate the extant instruments and develop new ones ensuring consistent criteria for the assessment of the demand and supply-side of populism.

Our analysis of CSES database indicates limited correlation between populist attitudes and support for populist parties in 10 out of the 17 countries studied. Congruence is only partial in France and Lithuania (only some attitudinal populist dimensions are correlated with “populist” vote), and null in other countries, such as Brazil, Korea and Greece. The lack of congruence is particularly significant among non-European countries as we find “populist voters” to be more prone to endorse parties classified as “populist” in only two—i.e., USA and Australia—out of the seven cases studied. Although, these highly asymmetric results *per se* do not invalidate the populist attitudes survey or party populism assessment made by CSES, they may be an indication that some of the items and criteria used nowadays to measure populism may not be ideal for large-scale cross-country comparisons.

We demonstrate that several of the items employed in the CSES Module 5 to measure the demand-side could be either replaced or refined. Our graded response model (GRM) suggests that only two items related to the e*lite* dimension (pop2 and pop4) significantly help discriminate between “populist” and “non-populist” voters in the CSES Module 5, and that the *out-groups* and *democracy* items present flat information slopes. In line with other recent studies, we suggest the need of previous theoretical and empirical validation processes of items *via* expert consultation and statistical methods, such as confirmatory factor analysis (CFA) and item response theory (IRT) (e.g., Castanho Silva et al., 2020; Van Hauwaert et al., 2020). Pilot analyses, although increasingly frequent, are not always conducted in studies claiming to measure and comparing populism. Properly documented expert validations and empirical tests are of great help to improve the choice of dimensions, items, and wordings in populist attitudes surveys. We also suggest considering alternative methods to the test and operationalization of scales, as well as including new items to analyze dimensions of populism studied outside the dominant ideational approach—such as “intersection items” and items capturing the appeal of populist discursive and performative styles.

Populism is considered a complex phenomenon and, accordingly, an increasing number of authors adopt a multidimensional angle to operationalize it. Although, CSES applies a multidimensional logic when studying the demand-side, it does not use a multidimensional assessment of populism when it comes to the supply-side. This discrepancy can be another factor explaining the lack of correlation between populist attitudes and the probability of supporting populist parties in many of the countries studied. We find disparities between the CSES Module 5 and POPPA scores for parties. More importantly, in some cases we find a higher correlation between the CSES Module 5 measurement of populist attitudes and the POPPA assessment of parties' populism, than when using the former in both instances. This suggests potential methodological issues with the CSES supply-side assessment, at least in some countries, worth investigating further. A more explicit process of selection of experts, definition of evaluation criteria and validation of results, as those followed in POPPA should be considered in future supply-side studies.

Moreover, authors developing new scales of populist attitudes should also consider, and hint at, how the items and dimensions they integrate in their analyses of the demand-side can be captured in the studies on the supply-side. Establishing correlations between populist attitudinal items and variables such as affinity, support or vote for “populist” parties—as those conducted in this paper—is a relevant first step to understand the connection between demand and supply, but insufficient if we are interested in shedding light on the causal narrative and on whether populism is basically a “pull” or “push” phenomenon. It would be advisable to engage in a wider variety of analyses of political ideas, discourses, performances, and strategies, and as well as the appeal and emotions they provoke on citizens. A more fine-grained multidimensional analysis of the populist supply may help understand what attitudinal aspects trigger, or are triggered by, political entrepreneurs or media frames.

Finally, through an analysis of average marginal effects for each populist party, we illustrate how CSES, can be better suited to measure some varieties of populism than others. Overall, the CSES scale of populist attitudes serves to predict support for Western right-wing parties, but it struggles to identify supporters of left-wing populist parties. We demonstrate that some of the items which may not be good predictors of populist vote in general, become statistically correlated with support for either left-wing or right-wing populist parties in specific countries. This is not an exclusive problem of CSES, recent studies have found similar limitations in other well-established populism scales. But this serves as a reminder of how challenging can be creating a comparative fit-for-all parsimonious tool to reliably capture different varieties of populism across diverse political contexts.

In sum, this paper has argued that we cannot confidently predict support for populist parties based on the current measurements of populist attitudes, less so to establish whether the populist market is mainly driven by supply or demand forces. The issues encountered cannot be exclusively attributed to theoretical consideration—i.e., choice of core attributes and dimensions—but also to methodological problems. Thus, this paper invites populism scholars to take into consideration both sides of populism when creating new instruments, and to adopt consistent and explicit criteria in their empirical work. Some of the problems of congruence detected in this paper could be addressed if the data collected had a similar granularity/dimensionality. Still our analysis suggests reevaluating extant measurement tools and operationalization strategies, as well as exploring new populism attitudinal items and party assessment criteria. A more consistent and flexible approach to the study of the demand- and supply-sides of populism would facilitate the detection of design problems and help test some of the longstanding theoretical assumptions in this field concerning varieties of populism, the relative centrality of attributes/dimensions, and the connection between populist parties and their voters.

## Data availability statement

The original contributions presented in the study are included in the article/Supplementary material, further inquiries can be directed to the corresponding author.

## Author contributions

JR has led on the statistical analysis and JO on the theoretical implications. Both authors contributed to all sections in this article and approved the submitted version.

## Funding

This research has been possible thanks to the financial support of the Talento Program of the Comunidad de Madrid (Project Code 2018-T1/SOC-10152) https://gestiona3.madrid.org/quadrivium/convocatorias/home/talento and that of the Agencia Estatal de Investigación (Project Code PID2020-113182RA-I00) https://www.aei.gob.es/. JO is the recipient of both awards and Principal Investigator in both projects. Funders played no role in the study design, data collection, analysis or decision to publish, or prepare this manuscript.

## Conflict of interest

The authors declare that the research was conducted in the absence of any commercial or financial relationships that could be construed as a potential conflict of interest.

## Publisher's note

All claims expressed in this article are solely those of the authors and do not necessarily represent those of their affiliated organizations, or those of the publisher, the editors and the reviewers. Any product that may be evaluated in this article, or claim that may be made by its manufacturer, is not guaranteed or endorsed by the publisher.

## Footnotes

^1^A search on Web of Science database shows that 77 academic documents were published in 2010 containing the terms “populism” or “populist” in their title and 860 in 2020.

^2^Some authors problematize the degree and nature of inclusion/exclusion discourses and classify populist parties according to them (Mudde and Rovira-Kaltwasser, 2013; De Cleen and Stavrakakis, 2017; Ibsen, 2019; Font et al., 2021).

^3^The construct validity and ability to capture multidimensionality has been questioned (Meijers and Zaslove, 2021, p. 374–375).

^4^The PopuList classifies political parties under the labels of populism, far-right, far-left and/or Eurosceptic (1 = if the party belongs to this label; 0 = if the party doesn't belong to).

^5^The question wording: “Parties can also be classified by their current use of POPULIST OR PLURALIST rhetoric. POPULIST language typically challenges the legitimacy of established political institutions and emphasizes that the will of the people should prevail. By contrast, PLURALIST rhetoric rejects these ideas, believing that elected leaders should govern, constrained by minority rights, bargaining and compromise, as well as checks and balances on executive power.” Norris (2020, p. 10). Where would you place each party on the following scale? From 0 (“Strongly favors pluralist rhetoric”) to10 (“Strongly favors populist rhetoric”).

^6^CSES Module 5 also contains items referring to other variables such as political interest, politics in the media, internal efficacy, attitudes toward redistribution, government performance, state of the economy and vote choice (Hobolt et al., 2016, p. 12).

^7^CSES Module 5 includes another item related to a general perception of corruption among politicians (Q7) but we consider that its wording makes it a weaker item to predict individuals' populist attitudes.

^8^It is worth noting that CSES Module 5 included other items for this dimension Q5b-d and Q6b-d, but for simplicity's sake we have only used the first of each of these broader questions. We previously checked the correlation between the different questions. Regarding the battery of questions of national identity (Q6a, Q6b, Q6c and Q6d) the correlation coefficient is major than 0.4 for all pairs of correlations with the exception of Q6d, related to country's customs and traditions. Question Q6a was selected for our analysis given that it presented the best fit among these 4 questions. The battery of questions of *out-groups*, displays different approximations to the attitudes toward immigrants, focusing on the effects of immigrants on the country's economy (Q5c), the country's culture (Q4d), and the country's crime levels (Q5e). The item selected (Q5a) focuses on the right of minorities to keep their customs and traditions. Q6b, although also referring to minorities (“The will of the majority should always prevail even over the rights of minorities”), it seems to introduce a majoritarian or people centric component which may overshadow the minority component.

^9^The exact wording is: “Please indicate the degree to which each of the parties can be characterized as a populist party. Populism can be defined as a thin-centered ideology that pits a virtuous and homogeneous people against a set of elites and dangerous “others” who are depicted as depriving the sovereign people of their rights, values, prosperity, identity, and voice. The emphasis on anti-elite/anti-establishment rhetoric and the contrast between the “pure people” and the “corrupt elite” are thus indications of the degree to which a party is populist. Populist parties may be found across the left-right ideological spectrum. On a scale of 0 to 10, where 0 is “not at all populist” and 10 is “very populist”, where would you place each of the parties?”.

^10^CSES published online a Macro Report for each of the countries available at https://cses.org/data-download/cses-module-5-2016-2021/. These reports contain the assessment of experts on populism and other areas of the study.

^11^We have excluded Chile due to data inconsistencies. All parties score 1, except for *Partido Progresista* which has a populism score of 2. Macro Report available at https://cses.org/datacenter/module5/macro/CHL_2017_Macro.pdf.

^12^CSES Module 5 assessment did not allow for decimals.

^13^No assessment of populism was made by CSES country experts on Taiwan and Turkey.

^14^The choice to dichotomize the level of populism in our analysis (1 = “populist party” −0 = “non-populist party”) used is not an unusual approach (e.g., Mudde, 2007; Rooduijn et al., 2019). It facilitates the visualization of the analyses conducted. Moreover, the Average Marginal Effects (AMEs) is a statistical technique which helps interpret the impact of each of the eight items on the likelihood of voting for a populist party rather than for other political options. It is based on a binomial logistic regression. Finally, a dichotomization seemed also the best option given that some CSES country expert reports did not include a score for some of the supposedly non-populist parties, this means that in the OLS regression analysis (see Figures A1, A2) we lost some cases.

^15^Pop1, pop2, pop3, pop4, pop5, pop6, and pop7 are Likert scales (1 = strongly agree; 2 = somewhat agree; 3 = neither agree nor disagree; 4 = somewhat disagree; 5 = strongly disagree) whereas pop8 is a categorical variable with four options (1 = very important; 2 = fairly important; 3 = not very important; 4 = not important at all). We create a dichotomous populist support variable (0 = casting a vote for a non-populist party; 1 = casting a vote for a populist party).

^16^Table A3 in the Appendix displays the statistical correlation between the eight populist items and Figure A7 shows the distribution in the responses to each of them.

^17^We divided by five the populism items in order to obtain variables ranging from 0 to 1 to facilitate the interpretation of the AMEs coefficients (this rescaling is neutral in terms of significance levels and does not affect the AMEs).

^18^We invert pop3 to make it follow the same (negative) direction than the rest of the items. However, it is worth noting that the compensatory approach implied by the additive index has been criticized by Wuttke et al. (2020), and it is also questioned later on in this paper.

^19^Rovira-Kaltwasser and Van Hauwaert (2020, p. 9) measure populist attitudes using items from previous scales and show surprising results. For instance, Mexico, which is ruled by a widely considered populist President, display very low average populist attitudes scores; much lower than countries such as France or Spain.

^20^Both techniques are very useful at survey development stage. While CFA examines covariances between different items, and constructs a linear response between the factor/dimension and item response, IRT assesses the overall response patterns across all items so that no information of the data is lost, and constructs a nonlinear relationship between latent traits and item responses (Depaoli et al., 2018, p. 1301; Embretson and Reise, 2000, p. 37–38).

^21^Given the continuous nature of the eight items to measure populist attitudes, we estimate Graded Response Models (GRM) instead of IRTs. GRM is based on a cumulative log-odds principle, in which the probabilities of choosing a given response category within each item is modeled as differences between cumulative probabilities (Van Hauwaert et al., 2020).

^22^Castanho Silva et al.'s (2020) CFA of the CSES scale suggests dropping the worst indicator.

^23^Still some discrepancies can be observed in the classification of parties across between these arguably more systematic and granular classificatory approaches.

^24^Taggart and Pirro (2021) consider that there is no populist party in Romania, Malta and Ireland so the score for these countries is 0.

^25^We classify parties as right or left-leaning in this analysis based on the average left-right self-placement by the citizens that expressed their support for them. Please note that there are no data for pop1 in some countries.

^26^L.S.-X.A. (Golden Dawn), AN.EL. and E.K. on the right and Syriza and K.K.E. on the left. In addition to the sociodemographic control variables (age, educational level and gender) we include voter's left-right self-placement, in order to detect false correlations.

^27^Pop5 is not statistically significant for the case of right-with populist parties, having a negative and significant effect to understand the left-wing populist vote.
