# Supplementary material for: Recalibrating populism measurement tools: Methodological inconsistencies and challenges to our understanding of the relationship between the supply- and demand-side of populism
Source: Front Sociol. 2022 Oct 14;7:970043. doi: 10.3389/fsoc.2022.970043 (PMC9614256; doi:10.3389/fsoc.2022.970043)
Supplement: Supplementary file 1 [file Data_Sheet_1.docx]

**Online Appendix: Robustness Checks**

**Table A1. Logistic and linear regression of the pooled dataset with country fixed effects**

|  | Model 1 | Model 2 |
| --- | --- | --- |
|  | Logistic | OLS |
| *Elites* |  |  |
| pop2 | -0.25* | -0.06* |
|  | (0.12) | (0.03) |
| pop3 | 0.31* | 0.07** |
|  | (0.10) | (0.02) |
| pop4 | -0.24* | -0.06* |
|  | (0.11) | (0.02) |
| *Democracy* |  |  |
| pop1 | -0.14 | -0.03 |
|  | (0.08) | (0.02) |
| pop5 | -0.12 | -0.03 |
|  | (0.07) | (0.02) |
| pop6 | -0.22** | -0.05** |
|  | (0.07) | (0.01) |
| *Out-groups* |  |  |
| pop7 | -0.25 | -0.06 |
|  | (0.70) | (0.17) |
| pop8 | -1.16* | -0.28* |
|  | (0.56) | (0.12) |
| Controls | YES | YES |
|  |  |  |

Robust standard errors in parentheses

*** p<0.001, ** p<0.01, * p<0.05

Note: Number of observations for pop1: 16,229; pop2: 19,313; pop3: 19,257; pop4: 19,145; pop5: 19,130; pop6: 19,235; pop7: 19,252; pop8:18,985*.*

*Source:* Own elaboration based on CSES

**Table A2.** **Confirmatory** **Factor Analysis**

chi2(28) = 8291.47 Prob>chi2 = 0.0000

Note: In bold the dimensions that belong to each one of the factors: pop2, pop3, pop4 and pop6 belongs to factor 1, whereas pop1, pop5, pop7 and pop8 belongs to Factor 2. Pop 3 is the only item with opposite expected impact.

*Source:* Own elaboration based on CSES

**Table A3. Correlation matrix (CSES populism scale items)**

|  | pop1 | pop2 | pop3 | pop4 | pop5 | pop6 | pop7 | pop8 |
| --- | --- | --- | --- | --- | --- | --- | --- | --- |
| pop1 | 1 |  |  |  |  |  |  |  |
| pop2 | 0,3330* | 1 |  |  |  |  |  |  |
| pop3 | -0,1767* | -0,4155* | 1 |  |  |  |  |  |
| pop4 | 0,3102* | 0,5212* | -0,3658* | 1 |  |  |  |  |
| pop5 | 0,1302* | 0,0953* | 0,0401* | 0,1217* | 1 |  |  |  |
| pop6 | 0,2377* | 0,3165* | -0,1942* | 0,3291* | 0,0748* | 1 |  |  |
| pop7 | 0,0822* | 0,1276* | -0,0259* | 0,1470* | 0,1816* | 0,0803* | 1 |  |
| pop8 | 0,1889* | 0,1936* | -0,1244* | 0,2063* | 0,1260* | 0,1693* | 0,1343* | 1 |

p-values < 0.05


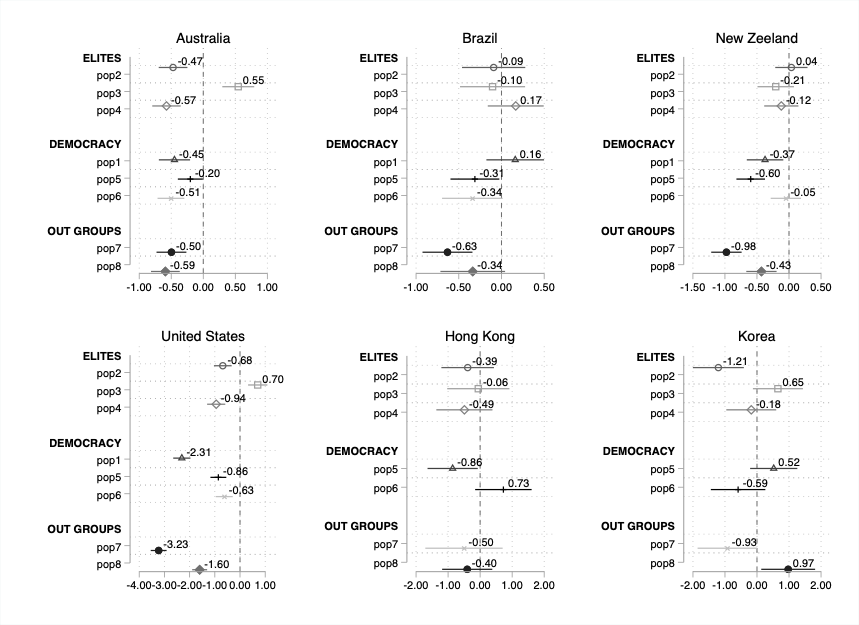


**Figure A1.** Ordinary least square, vote for populist parties in non-European countries.

**
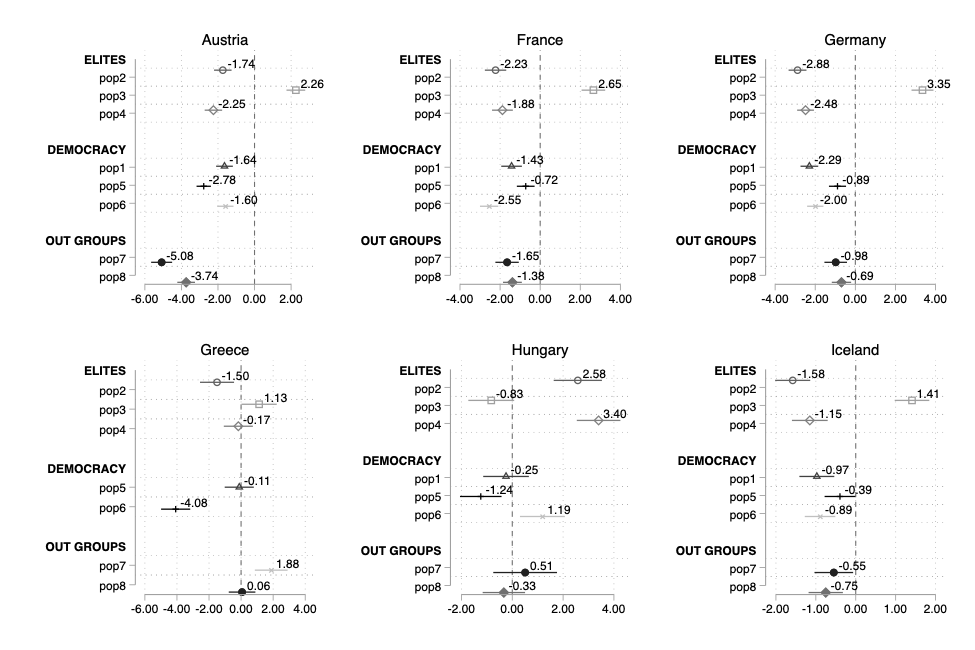
**

**
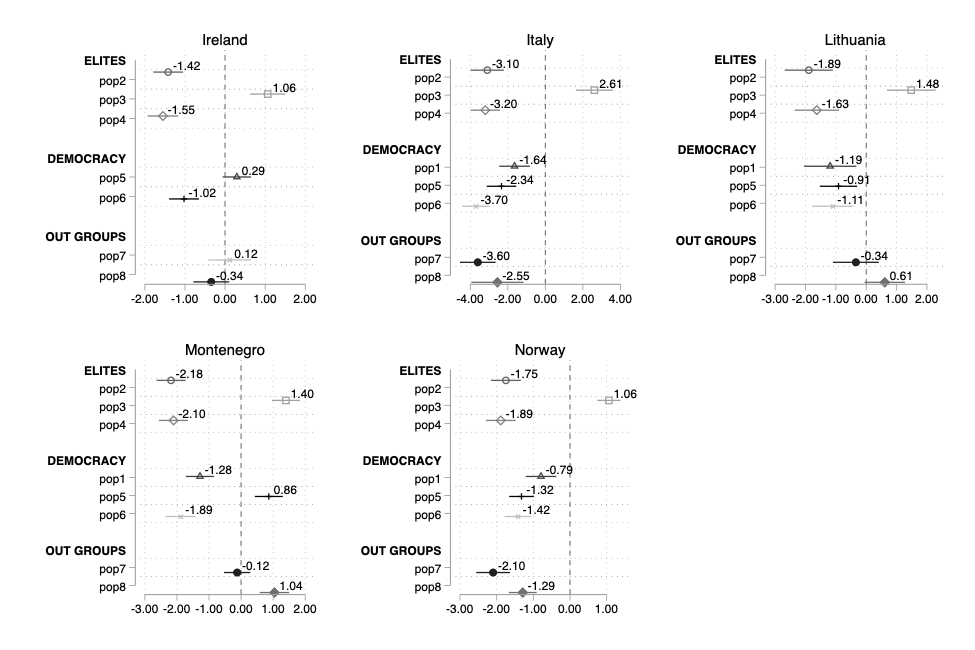
**

**Figure A2.** Ordinary least square, vote for populist parties in European countries.

Note: negative values of *pop_index* means major support to populist stances than to *pluralist* options

**Figure A3.** Margins plot for the relationship between the populist index and support for populist options (continuous variable), non-European countries**.**

Note: negative values of *pop_index* means major support to populist stances than to *pluralist* options

**Figure A4.** Margins plot for the relationship between the populist index and support for populist options (continuous variable), European countries.

**Figure A5.** AMEs specific populist (right) parties.

**Figure A6.** AMEs specific populist (left) parties.

**Figure A7.** Histograms of response categories, per item
